# Increasing Importance and Costs Associated With Publishing for Dermatology Residency Applicants

**DOI:** 10.7759/cureus.67520

**Published:** 2024-08-22

**Authors:** Hafsa Z Zuberi, Nathan Steele, Jonathan Aldrete, Cloyce Stetson

**Affiliations:** 1 School of Medicine, Texas Tech University Health Sciences Center, Lubbock, USA; 2 Department of Dermatology, Texas Tech University Health Sciences Center, Lubbock, USA

**Keywords:** medical education, research, scholarly activity, residency applications, dermatology

## Abstract

The financial costs associated with publishing in academic journals have steadily risen in recent years, reflected by higher publishing fees and the emergence of open access (OA) publishing models. Research remains an essential part of academia and has special significance for residency applicants. Due to recent changes in some objective measures used to rank residency applicants, such as abandoning numerical United States Medical Licensing Examination (USMLE) Step 1 scores and transitioning pre-clinical grades to Pass/Fail, other objective measures have gained significance: in particular, the quality and quantity of research activities including manuscripts, abstracts, and presentations have become more important in residency applications. This has led to a significant increase in the reported number of research experiences and publications to more competitive specialties, including dermatology. Our study analyzes the current financial landscape of publishing in the field of dermatology and the financial burden placed on applicants as well as programs to meet the expected number of research experiences in order to successfully match into a dermatology residency. Through a comprehensive examination of 85 dermatology-based academic journals, we assess the costs and differences of publishing in OA and hybrid OA journals while also exploring potential avenues for mitigating the financial burden of publishing. Our findings indicate that while cost-effective options exist, the financial burden of article processing charges remains substantial.

## Introduction

Dermatology remains one of the more competitive specialties for residency applicants to match into [1]. In addition to top percentile standardized test scores in the United States Medical Licensing Exam (USMLE) Step 1 and 2 and the National Board of Medical Examiners (NBME) Subject Exams/Shelf Exams, applicants must display a longitudinal interest in dermatology through avenues such as volunteer experiences, shadowing, leadership positions, and research publications [1]. With the transition of the USMLE Step 1 exam to Pass/Fail only, along with many medical schools changing pre-clinical grades to a Pass/Fail scoring system and the cancellation of the USMLE Step 2 Clinical Skills exam in the last five years, medical students and residency programs alike have shifted their focus toward other areas of the application to distinguish applicants in highly competitive fields such as dermatology.

As objective measures previously used to stratify applicants are abandoned, others have become increasingly employed; in particular, research experiences and meaningful publications have become more important in residency applications. However, this increased emphasis on research is complicated by the significant financial burden associated with publishing as well as varying institutional access to research opportunities. The introduction and widespread use of open access (OA) and article publishing charges (APC) have placed an undue financial burden on the authors, rather than the readers, to cover the cost of publication. Currently, most journals either employ an OA-only model in which all articles that are published require an APC and are immediately freely accessible, or a hybrid OA option. Hybrid OA journals offer two options for authors: green and gold. The green OA option does not charge an APC and is free for authors to publish but requires a subscription to the journal to read the article. The gold OA option requires the authors to pay an APC and the article is immediately and freely accessible to the public upon publication, similar to the OA-only model [2]. This study aims to explore the different publication options and investigate the impact of financial burdens on dermatology residency applicants associated with publishing research articles.

## Materials and methods

The metrics of dermatology-focused academic journals including publishing model and costs were analyzed in order to determine how they can affect medical students’ research activities and ultimately play a role in whether an applicant successfully matches to a dermatology residency program. We performed a systematic search of the directory of open access journals (DOAJ) and SCImago Journal Rank (SJR) list using the keyword “dermatology” to identify the relevant journals. The inclusion criteria were dermatology-based journals that accept English manuscripts, publicly display an APC on their website, and have an SJR and H index. The exclusion criteria for this study were journals that do not primarily publish articles in English and those that do not have publication metrics such as the SJR, H index, or APC publicly available. We individually analyzed each journal to determine if it met the inclusion criteria, resulting in 85 qualified journals included in the study. For each qualifying journal, we gathered the following metrics: SJR and SJR quartile, H index, and APC data (Table 1).

**Table 1:** Dermatology Journal Metrics A list of all journals included analyzed with metrics including their associated APCs (Article processing charge), H indices, SJRs (SCImago journal rank), and quartiles [2,3]. JDDG: Journal der Deutschen Dermatologischen Gesellschaft; JMIR: Journal of Medical Internet Research; JEADV: Journal of the European Academy of Dermatology and Venereology

Journal	Publication Model	APC	H Index	SJR	SJR Quartile
JAMA Dermatology	Hybrid	$5,000	179	2.766	Q1
British Journal of Dermatology	Hybrid	$3,416	194	2.254	Q1
American Journal of Clinical Dermatology	Hybrid	$4,890	101	2.072	Q1
Journal of the American Academy of Dermatology	Hybrid	$3,900	229	1.621	Q1
Journal of the European Academy of Dermatology and Venereology	Hybrid	$4,390	123	1.51	Q1
Journal of Investigative Dermatology	Hybrid	$3,400	220	1.435	Q1
Journal of Dermatological Science	Hybrid	$3,730	104	1.118	Q1
Psoriasis: Targets and Therapy	Open Access	$2,390	5	1.066	Q1
Mycoses	Hybrid	$4,330	81	1.059	Q1
Pigment Cell and Melanoma Research	Hybrid	$4,530	114	1.053	Q1
Dermatology and Therapy	Open Access	$6,850	38	1.034	Q1
Dermatology	Hybrid	$3,600	102	0.989	Q1
Experimental Dermatology	Open Access	$4,740	107	0.934	Q1
Burns and Trauma	Open Access	$2,400	29	0.928	Q1
Acta Dermato-Venereologica	Open Access	$1,780	92	0.897	Q1
JAAD International	Open access	$2,575	9	0.889	Q1
Archives of Dermatological Research	Hybrid	$4,390	88	0.887	Q1
Dermatitis	Hybrid	$3,161	61	0.874	Q1
Journal of Dermatological Treatment	Open Access	$3,500	59	0.852	Q1
Journal of Investigative Dermatology Symposium Proceedings	Hybrid	$3,200	86	0.809	Q1
The Journal of Dermatology	Hybrid	$3,630	75	0.808	Q1
Melanoma Research	Hybrid	$3,885	77	0.74	Q1
Wound Repair and Regeneration	Hybrid	$3,400	126	0.737	Q1
Contact Dermatitis	Hybrid	$4,690	105	0.72	Q1
Melanoma Management	Open Access	$1,600	12	0.699	Q1
International Wound Journal	Open Access	$3,410	76	0.693	Q1
Journal of Cutaneous Medicine and Surgery	Hybrid	$0	52	0.673	Q1
Journal of Dermatologic Therapy	Open Access	$2,500	77	0.667	Q1
International Journal of Women’s Dermatology	Open Access	$1,600	26	0.664	Q2
Journal of Drugs in Dermatology	Hybrid	$0	69	0.648	Q2
Clinics in Dermatology	Hybrid	$3,920	101	0.636	Q2
Clinical, Cosmetic, Investigational Dermatology	Open Access	$2,836	46	0.624	Q2
International Journal of Dermatology	Hybird	$3,760	101	0.623	Q2
Journal of Cosmetic Dermatology	Open Access	$2,500	55	0.61	Q2
Skin Research and Technology	Open Access	$2,200	77	0.577	Q2
Clinical and Experimental Dermatology	Open Access	$2,836	85	0.577	Q2
Journal of Cutaneous Pathology	Hybrid	$3,240	82	0.574	Q2
Journal of Tissue Viability	Hybrid	$2,980	38	0.572	Q2
Journal of Dermatologic Surgery	Hybrid	$3,806	134	0.56	Q2
Dermatologic Surgery	Hybrid	$4,532	134	0.56	Q2
Photodermatology, Photoimmunology & Photomedicine	Hybrid	$4,120	68	0.559	Q2
Skin Pharmacology and Physiology	Hybrid	$3,600	83	0.534	Q2
Pediatric Dermatology	Hybrid	$3,550	80	0.525	Q2
Cosmetics	Open Access	$2,315	35	0.509	Q2
Australasian Journal of Dermatology	Hybrid	$3,810	59	0.504	Q2
JDDG	Hybrid	$4,070	69	0.486	Q2
Journal of Clinical and Aesthetic Dermatology	Open Access	$0	49	0.473	Q2
International Journal of Cosmetic Science	Hybrid	$3,400	72	0.467	Q2
Dermatologica Sinica	Open Access	$0	20	0.463	Q2
Anais Brasileiros de Dermatologia	Open Access	$1,700	54	0.449	Q2
JAAD Case Reports	Open access	$675	27	0.44	Q2
Skin Appendage Disorders	Hybrid	$3,000	21	0.432	Q2
European Journal of Dermatology	Hybrid	$1,000	79	0.412	Q2
Dermatology Research and Practice	Open Access	$925	34	0.403	Q2
Journal of Cosmetic and Laser Therapy	Hybrid	$3,500	54	0.39	Q3
Advances In Skin & Wound Care	Hybrid	$4,141	68	0.389	Q3
Postepy Dermatologii I Alergologii	Open Access	$500	33	0.384	Q3
Journal of Psoriasis and Psoriatic Arthritis	Hybrid	$0	6	0.359	Q3
Dermatology Practical and Conceptual	Open Access	$0	6	0.353	Q3
Journal of Skin Cancer	Open Access	$925	13	0.35	Q3
Dermatology Online Journal	Open Access	$300	47	0.332	Q3
American Journal of Dermatopathology	Hybrid	$3,161	77	0.329	Q3
Indian Journal of Dermatology	Open Access	$0	46	0.318	Q3
Cutis	Hybrid	$0	58	0.317	Q3
Indian Journal of Dermatology Venerology and Leprology	Open Access	$0	52	0.314	Q3
Current Dermatology Reports	Hybrid	$3,860	18	0.303	Q3
Case Reports in Dermatology	Open Access	$1,080	21	0.288	Q3
Leprosy Review	Open Access	$0	48	0.285	Q3
Case Reports in Dermatological Medicine	Open Access	$725	6	0.282	Q3
Acta Dermatovenerologica alpina, pannonica et adriatica	Open Access	$280	29	0.269	Q3
Indian Dermatology Online Journal	Open Access	$0	5	0.25	Q3
Journal of Cutaneous and Aesthetic Surgery	Open Access	$0	19	0.244	Q3
Dermatology Reports	Open Access	$560	13	0.229	Q3
International Journal of Dermatology and Venereology	Open Access	$0	6	0.215	Q3
Iranian Journal of Dermatology	Open Access	$0	10	0.214	Q3
JMIR Dermatology	Open Access	$0	5	0.213	Q3
Annales de Dermatologie et de Venereologie	Hybrid	$2,500	39	0.207	Q3
SKIN The Journal of Cutaneous Medicine	Open Access	$0	5	0.19	Q4
Indian Journal of Leprosy	Open Access	$0	22	0.18	Q4
The Open Dermatology Journal	Open Access	$1,005	11	0.173	Q4
Open Dermatology Journal	Open Access	$1,005	11	0.173	Q4
JEADV Clinical Practice	Open Access	$2,700	5	0.173	Q4
Journal of Cutaneous Immunology and Allergy	Open Access	$2,800	5	0.173	Q4
Surgical & Cosmetic Dermatology	Open Access	$0	12	0.138	Q4
Turkderm Turkish Archives of Dermatology and Venerology	Open Access	$0	10	0.138	Q4

We additionally gathered data on which qualifying journals were indexed on PubMed by using the National Library of Medicine catalog [3].

SJR and H index data, both commonly used numeric measurements of journal prestige, were compiled on July 11, 2023, from the SJR database [4]. SJR is calculated by taking the weighted number of citations of a journal in a given year to citable publications published in the journal within the three preceding years, divided by the total number of citable publications published in the journal within the three preceding years. In regards to quartile, the top 25% of calculated SJR values are placed in quartile 1, the next 25% of calculated SJR values are placed in quartile 2, and so forth. H index is calculated by finding the maximum value of h given by a journal that has published at least h papers that have been cited at least h times [4]. A higher H index signifies that a journal (or individual author) has had a greater scholarly output that has been cited by other researchers; it can be used to compare publications to citations. We additionally identified the APCs for each journal on their individual websites. If a journal did not charge an APC, the APC for that journal was set at $0 for the purposes of our analysis. All APCs listed in currencies other than USD were converted to USD on July 11, 2023.

Journal metrics were analyzed using Microsoft Excel. We calculated the average APC, SJR, and H index for OA journals, hybrid journals, and all journals included in the study. Two-sample t-tests were used to compare the mean APC, SJR, and H index between OA and hybrid OA journals. Linear regression was used to calculate the correlation between APC and SJR.

## Results

**Figure 1:**
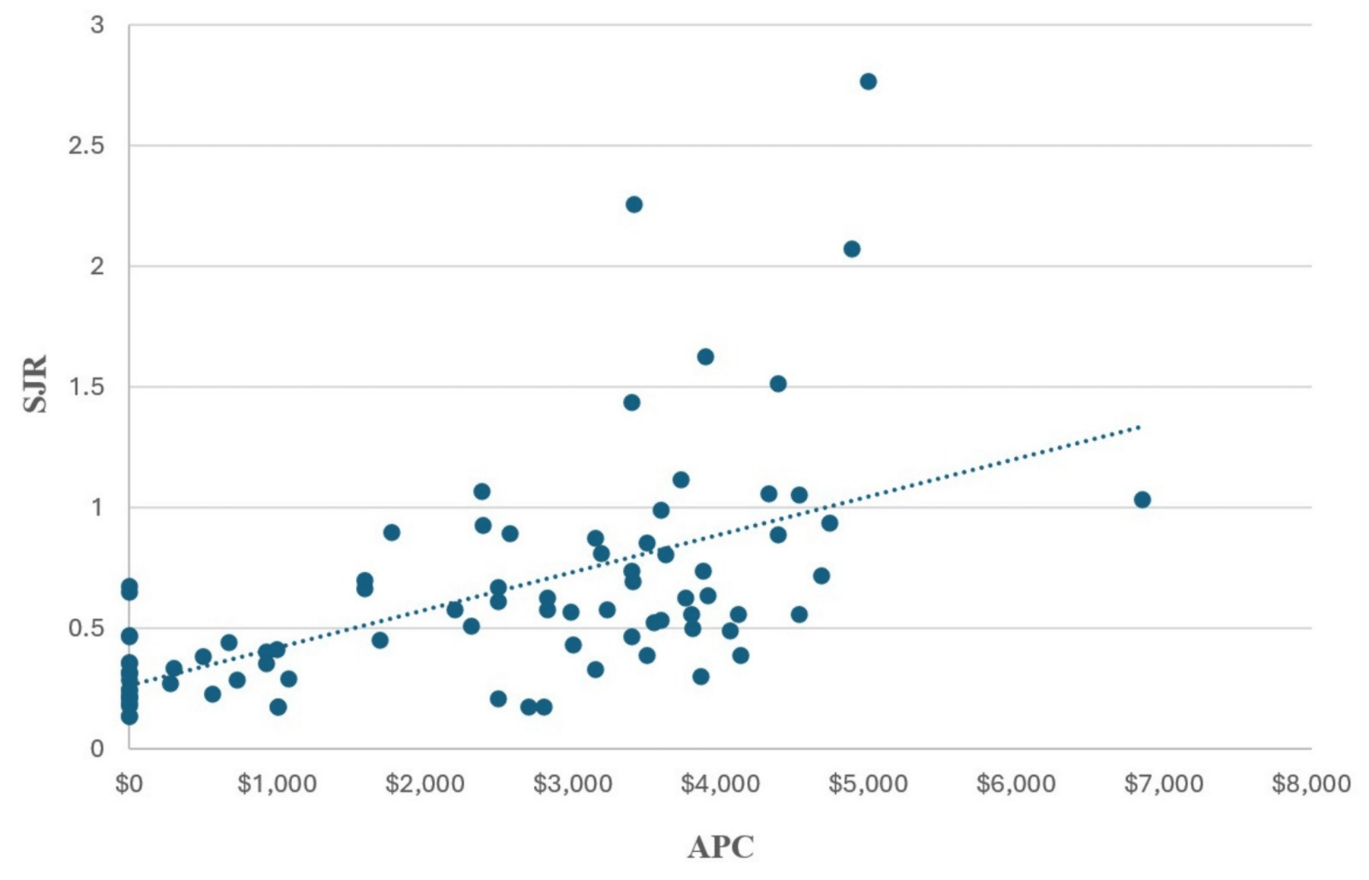
Comparison of Journal SJR to Journal APC SJR: SCImago journal rank; APC: article processing charges

**Figure 2:**
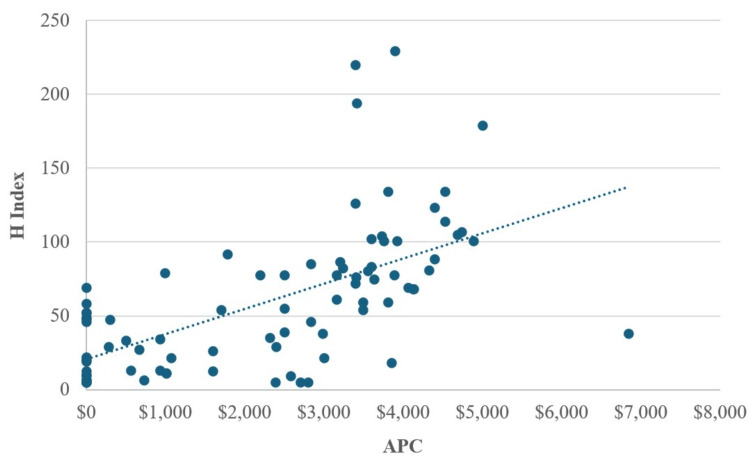
Comparison of Journal H Index to Journal APC APC: Article processing charges

Our study included a total of 85 journals, with 45 classified as OA journals and 40 as hybrid OA journals. APCs for these journals varied widely (ranging from $0 to $6,850) with 19 journals not charging an APC. The combined average APC was $2,290.64 while the average H Index was 59.75, and the average SJR was 0.622. The analysis of APCs compared to H Index and SJR is shown in Figures 1, 2.

APC compared to H index showed a statistically significant positive correlation between the two (r = .605, N = 85, p<0.001). APC compared to SJR similarly showed a statistically significant positive correlation (r = 0.584, N = 85, p<0.001).

An analysis of OA and hybrid OA journals separately yielded a significant difference in both publication quality and costs. The 45 OA journals included in our dataset showed an average APC of $1,360.27, an average H Index of 32.27, and an average SJR of 0.452, with 15 open-access journals not charging an APC. The analysis of the 40 hybrid OA journals revealed an average APC of $3337.30, an average H Index of 90.68, and an average SJR of 0.813, with four hybrid OA journals not charging an APC (Table 2).

**Table 2:** Average Metrics for Open Access Versus Hybrid Open Access Publication Models APC (article processing charges), SJR (SCImago journal rank), and H index for each group of journal publishing type. Formatted as mean (standard deviation) [2,3].

	Publication Type		Average APC	Average SJR	Average H Index
	All Journals		$2,290.64 (1717.73)	0.622 (0.461)	59.75 (48.298)
	Hybrid		$3,337.30 (1305.92)	0.813 (0.551)	90.68 (48.411)
	Open Access		$1,360.67 (1489.29)	0.452 (0.265)	32.27 (26.785)

Two-sample t-tests confirmed that the mean APC, SJR, and H index were all statistically different between OA and hybrid OA journals (P < 0.001). There was no significant positive or negative correlation found between APC and manuscript processing time or time from submission to first decision.

## Discussion

As the academic publishing landscape continues to evolve, the financial burden on residency applicants and programs continues to increase. If recent trends continue, preparing students for a successful match in fields dependent on high levels of scholarly activity such as dermatology can become increasingly expensive and difficult. According to 2022 National Resident Matching Program (NRMP) statistics, successfully matched applicants had an average of 20.9 publications, presentations, and abstracts and 7.2 research experiences [1]. These numbers are expected to rise as more students focus on producing publications to compensate for the absence of a numerical Step 1 score.

In addition to the quantity of applicants’ publications, the quality of those publications also plays a role. Publications in high-quality journals carry more weight in residency applications compared to those in lower-quality, pay-to-publish, or predatory journals. However, lower-ranked journals may be easier to publish in, thus attracting submissions from applicants who are actively trying to increase the number of research activities they have before submitting their residency application. One previous study showed that from 2007-2018, the number of publications per applicant in lower-impact journals increased four times faster than the number of publications in higher-impact journals [5]. Additionally, this number of publications may be inflated due to the Electronic Residency Application Service (ERAS) policy of allowing students to add “in progress” or “submitted” publications to their application [5].

Although publishing in highly ranked dermatology journals is more impactful for residency applications, it is also more expensive: our analysis revealed a statistically significant positive correlation between SJR and APC, indicating that publishing in higher-quality journals is both more challenging and more costly for programs. The average APC in this dermatology journal review was $2290.64. The financial burden may continue to increase as the number of publications necessary for a successful dermatology match continues to increase, and as more journals continue to shift to an OA-only policy. In institutions where research access and funding are limited, accommodating the interests of students interested in dermatology may be financially prohibitive and impractical. However, when students do publish in OA journals, their scholarly work may reach a wider audience: articles in hybrid journals generate 1.6 times more citations and four times more downloads than their counterparts in subscription-based journals [6].

Smaller academic and community programs are disproportionately impacted by trends in scholarly activity compared to larger institutions with more financial resources and more robust research infrastructure. The widening gap in publishing budgets and research access makes it increasingly difficult for applicants from smaller or community programs to distinguish themselves and successfully match to competitive specialties when compared to their peers in larger, well-funded programs. Attending a highly ranked (top 25) medical school also tended to correlate with a higher quantity and quality of peer-reviewed publications [5, 7]. As other objective measures are abandoned, otherwise capable applicants may be overlooked for lacking the number and quality of research activities expected. Aside from financial resources at a student's institution and school ranking, previously published studies show varying results on other demographic factors of applicants such as sex and geographic location and whether they yield increased or decreased number of publications, with no clear consensus being reached [6, 7].

Students lacking in research experience may elect to complete a research year while in medical school or following graduation to increase their research experiences and their chances of a successful match. Recent trends have shown that program directors of dermatology residency programs have given a demonstrated interest and involvement in scholarly activity a high score in regards to importance for residency applications [7]. However, research years are no guarantee of a successful match and are not without pitfalls. Often, research years will require relocation and can carry significant opportunity costs as well as other quotidian burdens, interrupting student loans, health insurance, and other benefits provided to medical students [8]. Though applicants who take a gap year for research have been shown to have up to double the amount of research projects on their residency applications, this does not always translate to a successful match [9]. Research years remain an untenable proposition to those from socioeconomic backgrounds that may not be able to bear the financial burden of relocation, and for whom the opportunity cost of passing on a year’s income/training without a guarantee of a successful match is too high. This further contributes to the inequity present within the medical field and undermines the effort for more equitable distribution of healthcare resources, including residency spots, to those from underrepresented socioeconomic backgrounds. 

Despite the rising costs, some affordable publication options still exist. We identified several OA journals in our dataset that do not charge an APC for publication. However, only one of these journals falls within the top quartile of journals according to SJR and only four are within the top 50% of journals. Publishing green OA in hybrid journals is another affordable option for authors, although it may reduce the article's impact compared to gold OA; nonetheless, publishing in these journals remains valuable for authors' residency applications. Though hybrid OA journals offer a financially friendly publication option, the transition to fully OA publishing is becoming more common among journals. One study analyzing orthopedic journals showed that the number of journals exclusively publishing OA articles has increased by more than 100% from 2012 to 2022 [10]. A similar trend may be seen in dermatology. As more hybrid OA journals switch to fully OA, cost-effective publication avenues will decrease.

The limitations of this study include disclosed and undisclosed discounts offered by journals to certain institutions or via preferred author programs, which we were unable to assess in our analysis. Additionally, this study is limited by the exclusion of some dermatology journals that did not meet the criteria. Furthermore, our study does not consider the difficulty of getting a manuscript accepted as part of our analysis. Future studies regarding this topic should focus on programs' budgets to support student-driven scholarly activity and how increasing publication costs affect them. Additionally, more research can be done on the impact of high-quality journals on application competitiveness. Finally, investigating the difference in article impact between green and gold OA publications can provide valuable insight.

## Conclusions

Scholarly activity is a vital portion of any candidate’s application for dermatology residency. However, rising costs can serve as a barrier to publication. This study highlights the financial burden resulting from the shift toward OA publication models. While cost-effective options still exist, APCs place significant strain on authors and their sponsoring programs, and as the illustrated trends continue, this financial burden will only increase. As the pursuit of research publications remains pivotal for success in dermatology residency applications, it is essential for medical schools, dermatology programs, and journals to address these escalating financial challenges to ensure equitable opportunities for aspiring dermatologists.
